# Editorial: Interaction between neuropsychiatry and sleep disorders: From mechanism to clinical practice

**DOI:** 10.3389/fneur.2022.1070040

**Published:** 2022-12-08

**Authors:** Bin Zhang, Shuqin Zhan, Junying Zhou, Xianchen Liu, Huajun Liang

**Affiliations:** ^1^Department of Psychiatry, Nanfang Hospital, Southern Medical University, Guangzhou, China; ^2^Xuanwu Hospital, Capital Medical University, Beijing, China; ^3^Sleep Medicine Center, West China Hospital, Sichuan University, Chengdu, China; ^4^Department of Neurology, West China Hospital, Sichuan University, Chengdu, China; ^5^Clinical Pharmacy and Translational Science, The University of Tennessee Health Science Center, Memphis, TN, United States; ^6^Diagnostic Radiology and Nuclear Medicine, University of Maryland School of Medicine, Baltimore, MD, United States

**Keywords:** sleep disorders, neuropsychiatric illness, insomnia, neurodegenerative disease, therapy

Sleep is a basic physiological need for human survival. While sleep problems are often the main reasons that drive an individual to seek medical treatment, conventionally, sleep problems have only been considered as symptoms of certain neuropsychiatric disorders, such as depression. However, increasing evidence suggests that sleep problems can also be a trigger for other neuropsychiatric disorders (1). Sleep is a physiological function that involves comprehensive regulating between the central neurological, metabolic, and immunological systems. Sleep disorders present as chronically interrupted quality, timing, amount of sleep, and impaired daytime functioning and may lead to neuropsychiatric illnesses (2). However, the mechanisms connecting sleep disorders and neuropsychiatric illnesses remain unclear. The current Research Topic represents a collection of papers that investigate the relationship between sleep disorders and neuropsychiatric illnesses, as well as studies analyzing the effects of some treatments for sleep disorders, particularly in the context of the COVID-19 pandemic.

As we all know, the COVID-19 pandemic has persisted for almost 3 years worldwide, and has increased stress, impacted mental health, and disrupted sleep for many people (3). During the COVID-19 pandemic, the prevalence of insomnia also increased (4). Specifically, situational insomnia, also called acute insomnia/short-term insomnia disorder, become more common in people who had no previous sleep problems (5). The article by Wu et al. finds that at 3 months post-COVID, those who had shorter rapid eye movement (REM) sleep latency and fragmented REM or NREM (non-REM) sleep also had more depressive symptoms (measured with Beck depression inventory score). Their findings indicate that REM sleep fragmentation may be a biomarker for depressive episodes in patients with short-term insomnia. Similarly, Qiao et al. find that impaired sleep quality and efficiency may be risk factors for depression and can predict the severity of depressive symptoms in 1,631 older Chinese adults.

Consistent with these studies that show sleep problems lead to mental health issues, Xie et al. find that those who had childhood trauma had poorer mental health during the pandemic, which is partly driven by their poorer sleep quality. Therefore, they suggest that mental health providers should pay more attention to individuals with childhood trauma, as childhood trauma is usually also associated with greater life stress and poor sleep. In a study conducted among college students, Wang, Liu et al. evaluate the association between chronotype and mental health. They find that compared to “early birds” or morning types, individuals defined as “night owls” or evening type were more likely to have long sleep compensation on weekends, insomnia symptoms, and depressive/anxiety symptoms. This study emphasizes that evening-type individuals should receive screening or intervention early due to their vulnerability to sleep or mental health problems.

The relationship between sleep disturbances and depressive/anxiety symptoms is further discussed in the articles by Xiao et al., Liang et al, and Wang, Liu et al. Using data collected from 12,178 college students in western north China, Xiao et al. find associations between depressive symptoms and insomnia were partly mediated by rumination. Whereas Liang et al. performed polysomnography (PSG) assessments and find that sleep perception affects sleep quality. In addition, they find that individuals with sleep misperception have common personalities and social behaviors, therefore, certain behavior treatments that target sleep misperception may help to improve their sleep and related anxiety symptoms. Last, immunologic (e.g., cytokine levels) and neurotransmitter systems (e.g., serotonin 2A receptor, 5-HTR2A) have important roles in the sleep-wake process (6, 7). The article by Wang, Gao et al. investigates the effects of cytokines and 5-HTR2A polymorphisms on sleep quality in non-manual workers in China. The results show that cytokines and 5-HTR2A polymorphisms not only have independent effects on sleep but may also cumulatively affect sleep quality.

Regarding treatments for sleep disorders, many studies used non-invasive brain stimulation techniques to reduce physiological arousal, a key component of insomnia (8, 9). Ma et al. perform a meta-analysis to evaluate the effectiveness of transcranial electric stimulation (TES) and repetitive transcranial magnetic stimulation (rTMS) in improving sleep quality. They find that rTMS shows a larger effect size than TES on improving objective measures of sleep including arousal, as well as on ameliorating subjective sleep complaints. Furthermore, Li et al. combine transcranial direct current stimulation (tDCS) and electroencephalogram (EEG) techniques to study sleep EEG complexity in patients with depression. They find that tDCS decreased intrinsic multi-scale entropy (which indicates improved sleep quality) during REM sleep without altering sleep structural integrity. The findings from Li et al. suggest daytime tDCS may be an effective method to improve sleep quality in depressed patients.

Acupuncture may be a safe alternative therapy for sleep problems, but the underlying mechanisms are unclear (9). In the systematic review and meta-analysis conducted by Zhao et al., acupuncture by itself or adjuvant to conventional pharmacotherapy (such as antidepressant and/or hypnotic) has low to moderate levels of evidence in treating insomnia patients with current depression. However, the benefit of acupuncture on residual insomnia in patients with remitted or partially remitted depression is limited. Also focusing on acupuncture treatment, Li et al. designed a randomized controlled trial to investigate the effectiveness of electroacupuncture in improving cognition after acute sleep deprivation and publish their protocol in this special issue.

CBTI remains the first-line treatment option for treating insomnia. In the article by Feng et al., one-week self-guided internet cognitive-behavioral treatments for insomnia (CBTI) improve insomnia symptoms and prevent situational insomnia from progressing to chronic insomnia during the COVID-19 pandemic. Xin et al. use bibliometric and visualization analysis to elucidate the trends of CBTI publication and show that the field of CBTI is maturing, with great study potential and broad prospects. They suggest future research should focus on creating new delivery models for CBTI that emphasize the prevention of insomnia and the scalability of treatments.

Obstructive sleep apnea (OSA) is a sleep disorder that characteristics by frequent arousal during sleep and excessive daytime sleepiness. Prajsuchanai et al. find that individuals with attention deficit hyperactivity disorder (ADHD) are prone to develop high-risk OSA. High-risk OSA was also associated with childhood obesity and affects children's quality of life, hence screen for high-risk OSA in children with ADHD may be cost-effective.

Primary restless legs syndrome (RLS), a less common sleep disorder, causes an intense, often irresistible urge to move your legs (sometimes arms or body) during sleep. The causes of PLS are still unknown. Liu et al. find that the patients with RLS have lower Vitamin D levels than healthy controls. Furthermore, they find that PLS patients with lower serum Vitamin D levels had worse sleep quality and more severe depressive symptoms. This study suggests a strong association between vitamin D and RLS that sheds light on developing more effective treatments for RLS.

Last but not least, sleep disorders have been well-established to be the early symptoms of neurodegenerative diseases, especially Parkinson's disease (PD). As described in the research by Yuan et al., REM behavior disorder (RBD) is not only a highly specific marker of PD but also one of the prodromal symptoms of PD. Yuan et al. studied the mechanisms that link RBD to PD and hypothesize that the TNF-α pathway might not be involved in disease progression from isolated RBD (iRBD) to PD by regulating the orexin system, although their results need to be verified further. Multiple system atrophy (MSA) is another neurodegenerative disorder characterized by both motor symptoms (Parkinsonism-like) and non-motor symptoms (excessive daytime sleepiness, EDS). Wang, Tang et al. find that in MSA patients, EDS mainly predicted mood and sleep-related breathing problems. For example, they find the severity of EDS is positively correlated with anxiety, depression, fatigue, and apnea-hypopnea index level. In addition, Yang et al. find that enlarged perivascular spaces (EPVS), an MRI marker of cerebral small-vessel disease, are associated with PD syndrome. EPVSs both in basal ganglia and in white matter contributed to poor sleep quality. This article also reviews the association between EPVS dynamic regulation, sleep-related neurotransmission, synaptic cleft metabolites clearance, and sleep-wake transition.

In summary, this Research Topic covers important aspects of sleep disorders and their connections with neuropsychiatric illnesses. We aim to update readers on the latest research findings in this field. These selected articles will provide insights for researchers in different fields into cutting-edge study methods in sleep medicine, thereby motivating multidisciplinary collaborations on elucidating underlying links between sleep disorders and neuropsychiatric illnesses, and ultimately, developing effective therapeutic strategies for both conditions.

## Author contributions

All authors have contributed intellectual content to the actual writing of the editorial. All authors contributed to the article and approved the submitted version.
